# Dissecting Allo-Sensitization After Local Administration of Human Allogeneic Adipose Mesenchymal Stem Cells in Perianal Fistulas of Crohn's Disease Patients

**DOI:** 10.3389/fimmu.2019.01244

**Published:** 2019-06-14

**Authors:** Alvaro Avivar-Valderas, Cristina Martín-Martín, Cristina Ramírez, Borja Del Río, Ramón Menta, Pablo Mancheño-Corvo, Maitane Ortiz-Virumbrales, Ángel Herrero-Méndez, Julián Panés, Damián García-Olmo, José Luís Castañer, Itziar Palacios, Eleuterio Lombardo, Wilfried Dalemans, Olga DelaRosa

**Affiliations:** ^1^Takeda Madrid, Cell Therapy Technology Center—Cell Therapies, Madrid, Spain; ^2^Department of Immunology, University Hospital Ramon y Cajal, Madrid, Spain; ^3^Department of Gastroenterology, Hospital Clínic, IDIBAPS, CIBERehd, Barcelona, Spain; ^4^Department of Surgery, Hospital U. Fundación Jiménez Díaz, Madrid, Spain; ^5^TiGenix NV, Leuven, Belgium

**Keywords:** ASC, cell therapy and immunotherapy, allogeneic, allo-sensitization, CD46, crispr gene editing, HLA class 1, complement dependent cytotoxicity (CDC)

## Abstract

Adipose mesenchymal stem cells (ASC) are considered minimally immunogenic. This is due to the low expression of human leukocyte antigens I (HLA-I), lack of HLA-II expression and low expression of co-stimulatory molecules such as CD40 and CD80. The low rate of observed immunological rejection as well as the immunomodulatory qualities, position ASC as a promising cell-based therapy for the treatment of a variety of inflammatory indications. Yet, few studies have addressed relevant aspects of immunogenicity such as ASC donor-to-patient HLA histocompatibility or assessment of immune response triggered by ASC administration, particularly in the cases of presensitization. The present study aims to assess allo-immune responses in a cohort of Crohn's disease patients administered with allogeneic ASC (darvadstrocel formerly Cx601) for the treatment of complex perianal fistulas. We identified donor-specific antibodies (DSA) generation in a proportion of patients and observed that patients showing preexisting immunity were prone to generating DSA after allogeneic therapy. Noteworthy, naïve patients generating DSA at week 12 (W12) showed a significant reduction in DSA titer at week 52 (W52), whereas DSA titer was reduced in pre-sensitized patients only with no specificities against the donor administered. Remarkably, we did not observe any correlation of DSA generation with ASC therapeutic efficacy. *In vitro* complement-dependent cytotoxicity (CDC) studies have revealed limited cytotoxic levels based upon HLA-I expression and binding capacity even in pro-inflammatory conditions. We sought to identify CDC coping mechanisms contributing to the limited cytotoxic killing observed in ASC *in vitro*. We found that ASC express membrane-bound complement regulatory proteins (mCRPs) CD55, CD46, and CD59 at basal levels, with CD46 more actively expressed in pro-inflammatory conditions. We demonstrated that CD46 is a main driver of CDC signaling; its depletion significantly enhances sensitivity of ASC to CDC. In summary, despite relatively high clearance, DSA generation may represent a major challenge for allogeneic cell therapy management. Sensitization may be a significant concern when evaluating re-treatment or multi-donor trials. It is still unknown whether DSA generation could potentially be the consequence of donor-to-patient interaction and, therefore, subsequently link to efficacy or biological activity. Lastly, we propose that CDC modulators such as CD46 could be used to ultimately link CDC specificity with allogeneic cell therapy efficacy.

## Introduction

The use of autologous and allogeneic mesenchymal stem cells (MSC) for treating a variety of conditions has been evaluated across several clinical trials (1), including those focused on the treatment inflammatory bowel disease (2), sepsis (3), and graft vs. host disease (GvHD) (4), as well as several autoimmune diseases (5). MSC therapeutic effects are largely attributed to their capacity to modulate immune response. MSC interact with both innate and adaptive arms of the immune system, inhibiting proliferation of T, B and natural killer (NK) cells and fueling expansion of regulatory T cells (Treg) (6, 7). MSC are able to hinder a series of immune cell functions, including: reducing cytokine secretion of T and NK cells; impairing maturation and activation of antigen-presenting cells (APC) and maturation and antibody (Ab) secretion of B cells (8, 9). Lastly, MSC have been shown to promote monocyte to macrophage transition and facilitate M1 (pro-inflammatory) to M2 (anti-inflammatory) macrophage skewing (10). Specifically, the immune modulatory capacities and therapeutic potential of allogeneic Adipose mesenchymal stem cells (ASC) have been extensively characterized by us and others (11–14).

The prevailing paradigm is that MSC in general, and ASC in particular, are not considered to induce a strong immunogenic response. This is mainly due to low human leukocyte antigen (HLA)-I expression, an absence of HLA-II expression, and a low expression of co-stimulatory molecules, such as CD40 and CD80 (7, 11). In fact, in the majority of clinical trials, patients have been administered with allogeneic MSC without the need of major histocompatibility complex (MHC) matching prior to treatment and without utilizing immunosuppressive therapies (15, 16), in contrast to other allogeneic cell-based therapies. Recent data has indicated that although well-tolerated, allogeneic MSC therapy has shown some signals of immune response, such as the generation of donor-specific antibodies (DSA) (17–21). Moreover, during allogeneic administration HLA-mediated cytotoxicity can potentially contribute to the elimination of ASC, eventually compromising efficacy. There are few studies investigating the immunogenicity of administered allogeneic ASC. The current study aims to address this issue.

Crohn's disease (CD) is a chronic inflammation of the digestive tract characterized by transmural inflammation and fistula formation (22). Perianal fistulas are a complication of CD that affects ~28% of patients in the first two decades following diagnosis (23). They are extremely difficult to treat as there are not any recognized effective treatment options, and the condition is associated with a high rate of relapse following the withdrawal of antibiotic or anti-tumor necrosis factor alpha (TNFα) therapy (22). ADMIRE CD (global randomized, double-blind, parallel-group, placebo-controlled trial) demonstrated that local allogeneic darvadstrocel (Cx601) administration can be an effective and safe treatment for complex perianal fistulas in adult patients with non- or mildly active luminal CD (21, 24). A cohort of ADMIRE CD patients was screened for generation of DSA, and it was determined that there were no safety signals associated with the development of DSA (24). A cohort of patients was screened for generation of DSA through Luminex solid-phase assays (SPA) using recombinant HLA molecules bound to beads.

The complement system is a key component of the innate immune response and serves as a nexus with the adaptive immune response, but it is primarily involved in inflammatory processes (25). During allogeneic recognition, complement-dependent cytotoxicity (CDC) is initiated when C1q, the initiating mediator of the classical complement pathway, is fixed to the Fc portion of HLA-I antigen-bound antibodies resulting in its activation and subsequent complement cascade signaling (26). Complement activation can be also triggered in damaged tissues where anaphylatoxins act as chemotactic factors to amplify the immune response. MSC express functional anaphylatoxin receptors (27) which facilitates their recruitment to the injured site and subsequent classic C1q CDC pathway activation. During the ADMIRE CD1 clinical trial, ASC were directly delivered into all fistula tracts and internal openings, which are essentially injured tissues (24). The prominent role of CDC in safeguarding homeostasis within damaged tissues (i.e., fistula) prompted us to examine the impact of the complement activation pathways in the fate of ASC.

Complement signaling is buffered by regulatory molecules (i.e., CD46, CD55, CD59) that are found in the membrane of different cell types, including ASC (28). These molecules function as decay accelerators and are known as membrane complement regulatory proteins (mCRP) (29). Despite mCRP expression being reported in MSC of varying origins, very little is known about their contribution to complement mediated cytotoxicity (30–32). Our objective is to evaluate the differential cytotoxic capacity among plasma samples collected from ADMIRE CD1 patients, using conventional functional assays against donor ASC *in vitro*.

## Materials and Methods

### Monitoring DSA Generation in ADMIRE CD

#### Patients

We focused in a subgroup of 123 patients enrolled in the randomized, double-blind, parallel-group, placebo-controlled study ADMIRE CD (24). All of them were adult patients (≥18 years) with CD and treatment-refractory, draining complex perianal fistulas and selected to a single local injection of 120 million ASC or 24 mL saline solution (control). A total of 60 and 63 patients received control or infusion of allogeneic ASC, respectively, from which 105 (58 ASC, 47 control) were successfully followed up 52 weeks after administration. Note, that clinical remission data was missing for 10 naïve patients which explains why number of clinical remission data-points (33) differs to total number of naïve patients in the study (34).

#### ASC Donors and Bone Marrow (BM)-MSC

Human adipose tissue aspirates from healthy donors were processed as described elsewhere (35). For the present study we used ASC from seven different donors, DonA was the donor used for ADMIRE CD clinical trial. Additionally, we tested mCRP expression and CDC sensitivity to the following donors: DonB, DonC, DonD, DonE, DonF, and DonG, manufactured by Tigenix (Takeda Pharmaceuticals). All ASC donors comply with International Federation for Adipose Therapeutics (IFATS) and the International Society for Cellular Therapy (ISCT) identity and purity criteria (36). ASC culture has been described elsewhere (11). BM-MSC were purchased to Lonza and cultured following manufacturer instructions.

#### HLA Antibodies (Abs) Detection

A plasma sample was obtained by centrifugation of a peripheral blood tube with ethylenediaminetetraacetic acid (Vacutainer® spry-coated K2EDTA tubes, BD™), collected from all patients, at baseline, 12 and 52 weeks after control or ASC administration. HLA Abs were detected in a Luminex platform using a LabscreenMixed™ kit (One Lambda Inc.® Canoga Park, CA, US) according to manufacturer instructions. All samples with a signal >800 units of median fluorescence intensity (MFI) were considered positive, and donor specificities for HLA Abs were determined using Labscreen Single Antigen™ kit (One Lambda Inc.® Canoga Park, CA, US). All signals were normalized according to Quantiplex™ beads fluorescence and specificities > 20,000 units of standard fluorescent intensity were considered relevant. Qualitatively, we defined the HLA antibody titer as the resulting MFI sum of all the determinant beads of the HLA class I molecules included in the Labscreen Mixed kit. We will refer to pre-existing HLA Abs detected in patients before ASC administration as HLA Abs whereas donor ASC-induced HLA Abs will be referred as DSA.

#### HLA Typing

The assignment of HLA allele expressed in patients or ASC donors was determined over DNA samples obtained from peripheral blood sample or ASC pellets using chemagic DNA Blood250 KIT (PerkinElmer). After checking purity via examination of the A260/280 absorbance ratio, all samples with a DNA concentration of 20 ng/μl or more were tested by LABType®SSO assay (One Lambda, Canoga Park, CA) specifically for loci A, B, and C of HLA according manufacturer instructions. The characterization of the incompatibilities between patient and donor ASC were defined as an unshared, unique chain of polymorphic residues, using the algorithm HLA matchmaker hereafter referred to as *eplets* (37).

### Standardization of Flow Cytometry Crossmatch (FCXM) Binding With Recombinant HLA Abs (rHLA)

#### Standard Curves

We established the level of class I (DonA and DonB) and class II HLA (DonA) expression in the indicated ASC donors used, under basal conditions and pre-activated with interferon gamma (IFNγ) (3 ng/mL for 48 h). We stained 50,000 ASC with the PE (R-phycoerythrin) anti-human class I HLA Ab (clone W6/32) and Peridinin Chlorophyll Protein Complex (PerCP) anti-human class II HLA Ab (clone L243) (Becton Dickinson, Franklin Lakes, New Jersey, US) in increasing concentrations (from 0 to 15 ng/ml for clone W6/32, and 0 to 3 ng/ml for clone L243) and incubated 30 min (min) in the dark at room temperature (RT).

#### Plasma Samples FCXM Binding Strength and CDC

We tested pre-treatment, week 12 (W12) and week 52 (W52) plasma samples of all patients who had received the ASC administration, previously de-complemented at 56°C for 30 min and washed once with autoMACS Running Buffer (Miltenyi). We incubated 50 μl of de-complemented plasma with 50,000 ASC in a final volume of 100 μl during 30 min at RT. Without washing we added 250 μl of rabbit serum as source of complement anti-human class I HLA (CABC-1D, One Lambda Inc.® Canoga Park, CA, US) for 1 h. Then cells were washed twice and incubated with 20 μl FITC anti-human IgG during 20 min and once washed, adding 5 μl of viability dye 7-Aminoactinomycin D (7-AAD) by acquisition in a LSR Fortessa flow cytometer (BD™). The presence of HLA antibodies and the recognition of ASC was determined by the increase of MFI with respect to the control serum and the cytotoxic capacity by the percentage of 7-AAD+ cells acquiring 10,000 events in P1 gate (total population of ASC) per sample. For analysis we used FlowJo software version 9.7.5.

### mCRP Quantification *via* FACS

ASC were grown in normal or 3 ng/mL IFNγ conditions for 48 h. ASC were then trypsinized and counted for a final concentration of 50,000 ASC per 100 μL autoMACS Running Buffer (Miltenyi). For antibody staining we used CD46 (564253, BD), CD55 (MCA1614PE, Serotec), and CD59 (BRA-10G, Novus Biologicals) Abs and their respective isotypes as controls (IgG2a-APC, IgG1-PE, and IgG2b-PE from BD). After 20 min ice incubation ASC were washed with autoMACS Running Buffer (Miltenyi) and centrifuged 500 × g for 4 min. Finally, ASC were resuspended in 100 μL autoMACS Running Buffer (Miltenyi) transferred to cytometry tubes acquired in a LSR Fortessa flow cytometer (BD) and analyzed with BD FacsDiva™ (BD).

### Generation of CD46^KO^ ASC

Guide RNA was designed to target CD46 exon 3 using the following public genomic tools: https://genome.ucsc.edu/, https://www.ncbi.nlm.nih.gov/gene. For CRISPR RNA (crRNA) delivery we used the Alt-R® CRISPR-Cas9 System (IDT Integrated DNA Technologies) as per manufacturer instructions. Briefly, ASC were thawed and left overnight. Following this, we prepared and delivered ribonucleoprotein (RNP) complexes using Lipofectamine™ RNAiMAX (Thermo-Fisher). We mixed crRNA and trans-activating crRNA (tracrRNA) in equimolar concentration in a sterile micro-centrifuge tube at a final oligo duplex working concentration of 1 μM. Following 20 min at RT mix incubation, we added the transfection complexes to the culture plate before adding the ASC suspension. After 24 h we replaced the ASC medium and verified lipofection efficacy of labeled tracrRNA-ATTO^550^ ASC with fluorescence microscope.

## Results

### Long-Term DSA Presence in ADMIRE CD Treated Patients

**Figure 1:**
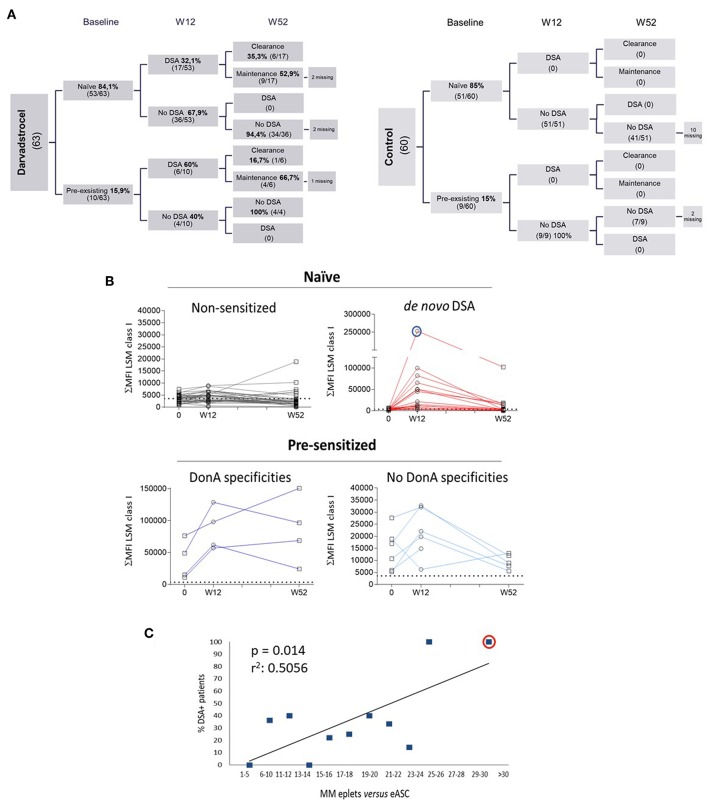
Characterization of DSA generation in ADMIRE CD. **(A)** Distribution of Labscreen Mixed/Labscreen Single Antigen (LSM/LSA) results for indicated visits: pre-treatment (baseline), week 12 (W12) and week 52 (W52) in both control (right chart) and darvadstrocel (left chart) arms of the study. A total of 5 (darvadstrocel arm) and 12 (control arm) patients withdrew from the study and, therefore, no LSM/LSA data were available. **(B)** Kinetic curves illustrating HLA Abs titer represented as the sum of MFI (ΣMFI) of each micro-sphere measured with the LSM assay at the indicated time points. The dotted line in each graph indicates the MFI > 3000 threshold applied for positivity. Pat92 is evidenced in a blue line circle. **(C)** Graph representing HLA incompatibility between each patient and ASC. For the correlation of incompatibility, the percentage of individuals that generated DSA was plotted vs. the number number (range) of mismatched *eplets*. Highlighted with a red circle is Pat92. For linear regression we applied Pearson test (*r*^2^). *P*-values were determined by the Student's *t*-test.

Blood samples were collected from 123 patients (63 ASC and 60 control) at baseline and 12 weeks after treatment administration. At 52 weeks after treatment administration, 105 patients (58 ASC and 47 placebo) provided blood samples (Figure 1A). Analysis by solid phase assay using Luminex technology revealed that 23 patients generated DSA 12 weeks after treatment. As expected, no patients receiving placebo generated DSA (Figure 1A, right chart). Additionally, results indicated that 16% (10/63) of treatment-ASC group and 15% (9/60) of placebo group had pre-existing HLA abs (pre-sensitized patients) at baseline. Out of the 53 naïve patients, 17 generated DSA at W12, and 6 of the 10 pre-sensitized patients generated DSA at W12 (Figure 1A, left chart). In all cases specificities of DSA were only detected against class I of HLA and not against HLA class II. Long term follow up revealed that no additional generation of DSA was detected 52 weeks after treatment and 7 out of the 23 sensitized patients (30%) cleared DSA at W52. Interestingly, the group of naïve patients generating DSA at W12 showed 35% clearance (6/17), whereas pre-sensitized patients generating DSA at W12 showed 17% (1/6) clearance at W52. Further, pre-sensitized patients were prone to sustained humoral response at W52 post-treatment, indicative for a sort of secondary response in pre-sensitized patients. In contrast, naïve patients generating DSA showed a trend to return to the initial baseline status as naïve patients, suggesting a primary immune response kinetic. The level of DSA positivity for a given sample was selected using the most restrictive threshold of the single antigen results, interpreted by categorical values (yes or no over a given cut-off). Alternatively, the amount of antibody bound relative to the total antigen present on the purified HLA-coated beads can be also quantified as the sum of MFI HLA class I LSM microspheres. We calculated time-course curves measuring plasma DSA titer throughout time (before treatment and 12–52 weeks post-treatment) illustrating the response kinetics and determining the likelihood of reducing their DSA levels (Figure 1B). Patients from the treatment group were clustered in the following groups: Naïve patients that did not generate DSA (where baseline levels will be used for comparisons), naïve patients that generated DSA after allo-ASC administration, pre-sensitized patients sharing specificities of the donor ASC administered and pre-sensitized patients not sharing specificities against the donor used. As expected, naïve patients not generating DSA did not modify their antibody titer throughout the course of the study (Figure 1B, upper left panel) whereas naïve patients that generated DSA exhibited an increase of antibody titer at W12 that reduces at W52, mimicking a primary immune response kinetic. In addition, baseline MFI values to W12 differed among patients, with certain patients exhibiting a particularly intense response, patient 92 (Pat92) being the most allo-reactive (Figure 1B, blue circle). Interestingly, the kinetics of the pre-sensitized group of patients that shared specificities with donor administered did not show a consistent reduction of antibody titer at W52 (Figure 1B, lower left panel), whereas those pre-sensitized patients that did not share specificities with the donor administered showed increase of DSA titer at W12 that is reduced at W52 (Figure 1B, lower right panel) like the primary immune response kinetics observed in the upper left. We examined a possible connection between donor–patient HLA matching grade and the probability to generate DSA (38, 39). We aimed to identify polymorphic residues present in the ASC donor HLA type (*eplets*) but absent in patients as main precursors of the allogeneic recognition. Each patient's HLA allele was aligned with ASC donor HLA allele for mismatch quantification (37). In our study, allo-sensitization arose mainly against HLA class I (*data not shown*), therefore we focused in characterizing loci A and B of HLA class I. Total number of *eplets* correlated with patients' susceptibility to generate DSA (Figure 1C).

Finally, we focused on investigating a potential connection of DSA generation and ASC therapeutic effect. We correlated DSA generation with clinical remission (closure of all treated external openings draining at baseline) at W12 and W52 (Supplementary Figure 1A). DSA presence did not affect clinical remission ratios in naïve patients (66.7 against 67.6%) at W12 clinical end-points. Clinical remission levels were slightly higher in naïve patients with DSA vs. patients that did not generated DSA (55.6 against 50%) at W52 end-point. However, no statistical difference was observed at this clinical end-point. Due to the low number of pre-sensitized patients, no statistical analysis could be performed in this sub-group.

To summarize, the above data suggest that although ASC could trigger allo-sensitization in a segment of patients, in most cases we observed a reduction in the antibody titer over time. Although we identified a link between DSA generation and donor-to-patient histocompatibility (based on HLA class I A and B loci mismatch) no correlation with clinical efficacy was observed.

### *In vitro* Binding of HLA Abs to ASC

**Figure 2:**
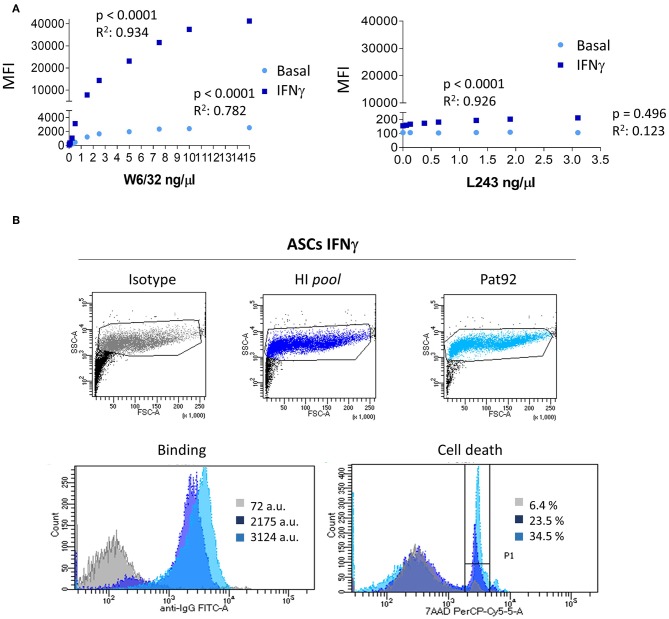
HLA expression in ASC and *in vitro* binding HLA Abs. **(A)** Graphs showing the correlation between MFI increase and each concentration of class I HLA (W6/32) Ab and class II HLA (L243) directed against untreated (light blue) or pre-activated with IFNγ (dark blue) ASC. **(B)** Plots of FcTox (complement-dependent cytotoxicity by flow cytometry assay) representing negative control (isotype), positive control (hyper-immunized samples, HI pool) and patient 92 (Pat92). Lower panels show histogram analysis of binding strength (left) and percentage of cell death, 7-AAD % (right panel). *P*-values were determined by the Student's *t*-test and *r*^2^ by Pearson test.

Although MSC found in different tissues share common hallmarks including immuno-modulatory properties or identity markers, differential immunogenic responses have been reported in *in vivo* models (40–44). To characterize immunogenic response of ASC we first sought to quantify the expression of HLA-I and -II molecules on DonA ASC and their ability to bind patient's HLA Abs *via* FACS analysis (Figure 2A). We incubated ASC, pre-stimulated or not with IFNγ, with increasing concentrations of fluorescence-labeled HLA-I (W6/32) or HLA-II (L243) recombinant Abs and quantified the MFI of the staining. As expected, ASC expressed HLA-I at basal level exhibiting a strong increase after IFNγ stimulation (Figure 2A). Conversely, HLA-II levels were negative at baseline and were increased following IFNγ stimulation.

Among all plasma samples analyzed, one interesting high HLA titer sample brought our attention to a detailed analysis. This plasma sample, Pat92 (Figure 1B, blue circle), was carrying the largest DSA titer post-treatment. Following IFNγ stimulation, we observed a significant increase of ASC binding strength exclusively in positive control (pool of hyper-immunized samples, HI *pool*) and Pat92 sample (Figure 2B, lower left panel). The increase in the binding was accompanied by high percentage of cytotoxic killing, specifically 34.5% in Pat92 (Figure 2B, light blue). This percentage was significantly higher than the percentage of killing quantified in the rest of 22 patients tested (ranging from 3.3 to 9.3%, *data not shown*), confirming that Pat92 was the most allo-reactive. Interestingly, Pat92 was the one showing highest level of mismatching with the ASC administered (37 mismatched *eplets*) and did clear antibody 52 weeks after treatment. Further studies will allow us to better understand whether there is a correlation between pre-sensitization and lack of efficacy due to the immediate ASC elimination.

### Plasma DSA Binds ASC Inducing Moderate Killing *in vitro*

**Figure 3:**
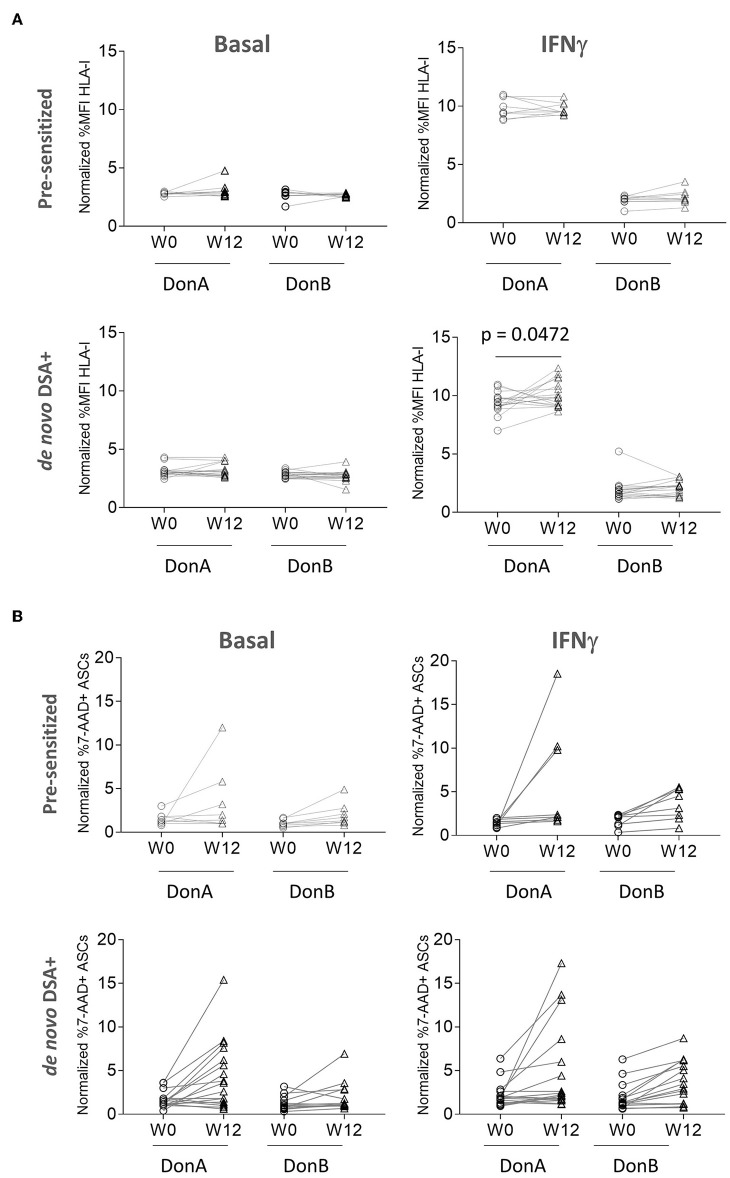
ADMIRE CD plasma samples induce low cytotoxic killing in ASC *in vitro*. **(A)** Graphs showing normalized percent values of HLA-I binding in 10 pre-sensitized (upper panels) and 17 *de novo* DSA+ patients (lower panels) at the indicated time-points (W0 pre-treatment and W12 post-treatment). Prior to binding assay DonA (donor administered in the ADMIRE CD trial) and DonB ASC were grown in normal (basal) conditions or in presence of 3 ng/mL IFNγ (IFNγ) for 48 h. **(B)** Graphs showing normalized percent values of 7-AAD positive ASC in 10 pre-sensitized (upper panels) and 17 *de novo* DSA+ patients (lower panels) at the indicated time-points. *P*-values were determined by the Student's.

In the results above we have shown that from a cohort of 63 ADMIRE CD patients, 10 had pre-existing HLA-I Ab and 17 generated DSA *de novo*. We have also demonstrated that ASC express HLA-I antigen and bind to rHLA-I Ab; however, it is unknown whether patients' DSA have the ability to bind and, subsequently, induce cytotoxic killing of ASC. To test that, we strove to quantify differential affinities of pre-sensitized and *de novo* DSA+ groups to bind HLA class I antigens in ASC *in vitro*. In addition to the original donor, DonA (administered in the ADMIRE CD trial), we included an additional donor, DonB (Supplementary Figure 1B), to function as a control for DSA specificity since it was not administered to the patients (Figure 3A). We analyzed baseline and W12 samples *via* FCXM and measured HLA-I binding strength to ASC cultured in basal conditions or pre-stimulated with IFNγ (Figure 3A, upper panels). As expected, we did not observe high binding capacity in pre-sensitized nor in *de novo* DSA+ patients samples for both ASC donors in basal conditions. When ASC were pre-stimulated with IFNγ, we observed that pre-sensitized patients showed high and comparable binding affinities in samples from W0 and W12 visits only with DonA (Figure 3A, upper panels). As expected, basal conditions and thus low HLA-I antigen expression in the membrane of ASC donors, correlated with low binding affinity both in DonA and DonB (Figure 3A, lower panels). Similarly, we detected a significant increase the binding affinity when we compared W12 vs. baseline in *de novo* DSA+ patient's samples only with DonA (Figure 3A, lower panels). These results agree with solid transplant flow cytometry cross-matching observations (33), where only patients with high DSA levels that also share immune-specificities with donors resulted in significant HLA-I binding.

Next, we aimed to understand whether pre-existing HLA-I Abs and DSA generated after allogeneic administration of ASC were able to fix complement, and therefore trigger *in vitro* CDC. In the cohort of pre-sensitized patients, W12 samples induced modest cytotoxic killing in DonB (0–5% 7-AAD+ cells) both in basal and IFNγ conditions. As expected, incubation of same samples induced higher cytotoxic killing in basal (2 patients) and IFNγ (3 patients) exclusively in DonA ASC (Figure 3B). Similarly, patients with high cytotoxic levels in basal conditions increased their threshold when stimulated with IFNγ at W12 (Supplementary Figure 2) exclusively in DonA ASC. *De novo* DSA+ W12 samples were able to fix complement and induce cytotoxic killing in basal conditions. As anticipated, a higher percentage of cell death was reached exclusively in DonA when ASC were stimulated with IFNγ (Figure 3B). At W12, IFNγ stimulation could increase cytotoxic levels even to patients with already high basal levels (Supplementary Figure 2) specially in DonA. This data suggests that pre-existing HLA-I Abs and DSA can bind ASC and induce modest CDC specifically in DonA ASC. Interestingly, although DonB did not induce significant cytotoxic percentages neither in pre-sensitized nor in *DSA*+ cohorts, we observed a trend of increased death levels in W12 vs. baseline. One possible explanation could be the existence of shared HLA polymorphic alleles between the two donors used in this study. To evaluate that we performed HLA typing and observed indeed that DonB shares the same HLA-A allele with DonA (Supplementary Figure 1B). We believe this shared HLA allele might be responsible for the trend toward increased cytotoxic levels in W12 samples in DonB.

### High Expression of mCRP in ASC

**Figure 4:**
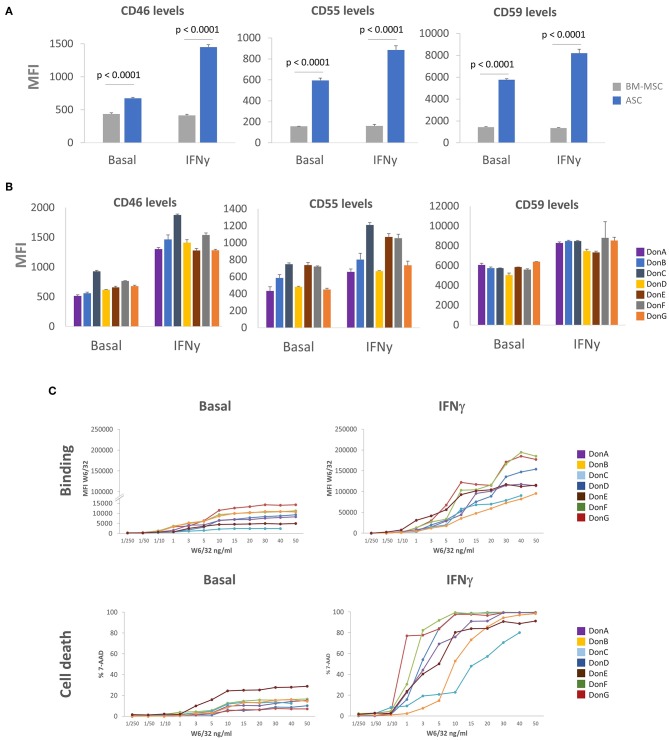
ASC express high levels of mCRP. **(A)** Graphs showing MFI values of CD46, CD55, and CD59 in seven ASC donors (blue bars) and one BM-MSC donor (gray bars) *via* FACS analysis. Cells were grown in the presence of 3 ng/mL (IFNγ) for 48 h or left untreated (basal). **(B)** Graphs showing differential MFI values of CD46, CD55, and CD59 in the seven ASC donors *via* FACS analysis. Cells were grown in the presence of 3 ng/mL IFNγ for 48 h (IFNγ) of left untreated (basal). **(C)** *P*-values were determined by the Student's *t*-test.

To understand the moderate killing of ASC impinged by pre-existing HLA Abs and DSA we sought to identify complement inhibition strategies that might enable ASC to cope and/or evade cytotoxic killing. One classical mechanism for complement signaling inhibition is the induction of mCRP proteins CD46, CD55, and CD59 (29, 45, 46). While some authors have shown that MSC express low levels of CD46 and CD55, and high CD59 (30), others suggest that MSC express moderate levels of all mCRP (31, 32). To address this controversy, we analyzed CD46, CD55, and CD59 expression levels in seven ASC donors stimulated or not with IFNγ, and compared expression levels with commercial BM-MSC (Figure 4A). We observed that basal levels of CD46, CD55, and CD59 were higher in ASC compared to BM-MSC. To recreate a physiologically relevant scenario, we tested mCRP levels in the presence of IFNγ (pro-inflammatory environment), which is also a critical mediator of ASC immune-modulatory response (11). We did not observe significant modulation of mCRPs in BM-MSC, whereas ASC appeared to potently induce mCRP following IFNγ stimulation. mCRP induction was particularly prominent in CD46 with a ~2.14-fold increase following IFNγ stimulation (Figure 4A). While the expression pattern of mCRP in different ASC donors was comparable, some donors exhibited differential expression of specific mCRPs (Figure 4B). Specifically, DonC exhibited higher CD46 and CD55 levels than rest of donors; DonC, DonE and DonF preferentially over-expressed CD55 (Figure 4B). This prompted us to investigate whether the differential expression of mCRP could impinge differential sensitivities to CDC. To answer this, we investigated the kinetics of HLA-I antigen expression and binding affinities among this panel of ASC donors. ASC donors were incubated with increased concentrations of recombinant HLA-I antibody (rHLA-I) W6/32 and measured its binding affinity with FACS (Figure 4C). We observed different binding affinities among donors in basal conditions (i.e., a 12-fold difference when comparing DonC with DonG with 10 ng/mL W6/32), suggesting differential antigen HLA-I expression. As expected, following IFNγ stimulation HLA-I antigen was induced in ASC donors as indicated by increased W6/32 binding (Figure 4C). Again, we observed varying binding affinities among donors (a 6.25-fold difference at 10 ng/mL comparing DonB with DonG). In parallel, we studied the sensitivity of the different ASC donors to the CDC assay. Under basal conditions DonE exhibited ~25% cell death at the highest W6/32 concentrations, in the rest of the donors this was ~15%, except DonD and DonG which exhibited a lower percentage (Figure 4C). As predicted, following IFNγ stimulation CDC sensitivity levels increased dramatically in all ASC donors, but to a lesser extent for DonB and DonC, that remained relatively resistant to CDC-mediated cell death (Figure 4C).

Next, we sought to determine whether high W6/32 binding affinity would correlate with enhanced CDC sensitivity. For performing this correlation analysis, we set W6/32 concentration to 10ng/mL as this was the Ab amount driving the transitional phase of the curves (after exponential and before plateau). We did not observe any significant correlation in basal conditions nor in the IFNγ stimulated scenario (Supplementary Figure 3A). Remarkably, we identified a group of ASC donors that despite expressing relatively low W6/32 Ab MFI levels (low binding) exhibited high sensitivity to CDC. This suggested that there is not an absolute correlation between W6/32 Ab binding and sensitivity to CDC. We hypothesized that ASC donor-specific expression of mCRP might be the main driver of CDC sensitivity among donors. To determine which of the three mCRPs is the major contributor to CDC inhibition, we correlated cell death levels reached with 10 ng/mL of W6/32 with MFI expression levels of the three mCRPs both in basal and IFNγ conditions (Supplementary Figure 3B). We did not observe a positive correlation in basal conditions with any of the tested mCRP molecules. However, we noticed that following IFNγ stimulation, lower CD46 and CD55 levels significantly correlated with higher cell death levels. Finally, CD46 slope significance was slightly higher compared with CD55.

### CD46 Depletion Increases CDC Sensitivity of ASC *in vitro*

The robust induction of CD46 following IFNγ stimulation compared to CD55 or CD59 and the higher significance of cytotoxicity correlation together with the reduced inter-donor variability of CD46 vs. CD55, prompted us to perform an in-depth analysis of CD46 and its potential impact in CDC sensitivity in ASC. Using public genome browsers, we identified top gRNA sequences to knock-down *CD46* (ncbi.nlm.nih.gov/gene and crispr.mit.edu). We selected optimal gRNA sequences based on two parameters, high specificity and low off-target score. Two optimal crRNAs (crRNA1 blue and crRNA2 yellow) targeting exon 3 were selected for efficacy screening (Supplementary Figure 3C). Delivery of crRNA:tracrRNA-ATTO^550^:Cas9 complexes was examined under fluorescence microscope. We observed that after 24 h of lipotransfection the vast majority of ASC had incorporated the RNP complexes which would correlate with high Cas9-mediated double-strand breaks events (Supplementary Figure 3C). To check CRISPR-mediated knock-down efficacy, we cultured ASC in the presence or absence of IFNγ and analyzed CD46 expression *via* FACS. The efficacy of crRNA1 was comparable to crRNA2 both in normal and IFNγ conditions, thus we selected crRNA1 for the generation of the ASC-CD46^KO^ clones (Supplementary Figure 3D).

**Figure 5:**
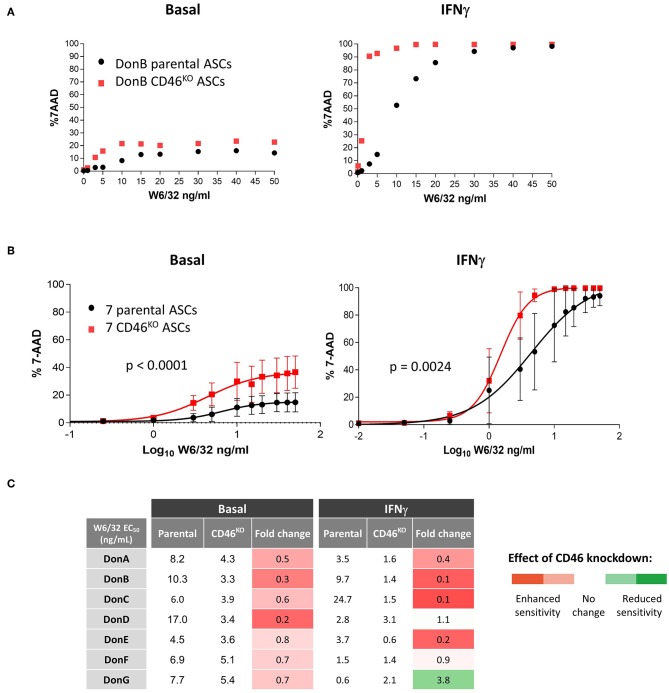
CD46 is a key mediator of complement cytotoxicity in ASC. **(A)** Graph showing percentage of 7-AAD positive parental and CD46^KO^ DonB ASC against increased concentration levels of W6/32 Ab. Prior analysis parental and CD46^KO^ DonB ASC were grown in the presence of 3 ng/mL IFNγ for 48 h (IFNγ) or left untreated (basal). **(B)** Sigmoidal curves displaying percentage of 7-AAD positive parental and CD46^KO^ ASC against EC_50_ of W6/32 Ab (transformed from lineal to log_10_). *P*-values were determined by the two-way ANOVA test. **(C)** Table showing half-maximal effective concentration of W6/32 Ab (ng/mL) of seven parental and the corresponding CD46^KO^ ASC in basal and IFNγ conditions. In columns 4 and 7, we calculated fold-change differences (CD46^KO^ EC_50_/parental EC_50_) and applied the color code showed in the right.

We then selected DonB having low CDC sensitivity to test whether selective depletion of CD46 could sensitize it to cytotoxic killing. The W6/32-mediated cytotoxic assays was performed in basal and IFNγ conditions (Figure 5A). As expected, CD46 knock-down induced modest increase in the cytotoxic killing in basal conditions (Figure 5A, left graph), which was likely due to the low HLA-I binding and subsequent complement fixation. Following IFNγ stimulation we observed a significant boost in the percentage of cytotoxic killing in parental ASC and this was further enhanced in CD46^KO^ ASC (Figure 5A, right graph). At a physiological dose of 10 ng/mL W6/32 we obtained ~50% 7-AAD positive cells in parental ASC and CD46 knock-down increased the percentage of killing up to ~95%, suggesting that CD46 plays a critical function in preventing cytotoxic killing.

To test whether CD46 cytotoxic inhibitory functions are effective in other ASC donors, we performed cytotoxicity analysis in the panel of seven donors and plotted mean curves in basal conditions (Figure 5B, left graph) and after ASC IFNγ stimulation (Figure 5B, right graph). Under both testing conditions CD46^KO^ ASC donors exhibited enhanced sensitivity to CDC than parental controls. A shift to the left of the half maximal effective concentration (EC_50_) curves implies a decrease in the concentration of the W6/32 Ab required to induce CDC, which correlates with enhanced sensitivity to CDC. Next, to quantify the shift in the curves we calculated of W6/32 Ab (Figure 5C). In basal conditions, CD46^KO^ donors exhibited decreased EC_50_ compared to parental donors, suggesting higher sensitivity to W6/32 (Figure 5C, fourth column). We observed a similar effect in IFNγ conditions (Figure 5C, seventh column), confirming our original hypothesis that *CD46* expression confers CDC resistance to ASC *in vitro*. This data confirms that *CD46* is a key mediator of CDC and that its depletion in ASC correlates with enhanced CDC sensitivity.

## Discussion

There are high expectations for utilizing allogeneic MSC as therapeutic tools for treating numerous diseases; however, further investigations are required to assess safety and potential toxicity. Few studies have examined their immunogenicity by characterizing the immune responses induced by their therapeutic administration. Noteworthy, a significant number of studies have found that MSC administered in several animal models with mismatching MHC are in fact rejected (40–42). These observations allow us to strongly argue against the canonical notion that MSC are immuno-privileged. Nevertheless, several clinical studies have demonstrated that allogeneic MSC treatment induces DSA without having negative consequences on safety or efficacy (19, 20, 47). In general, potential immunological risk has been downplayed based on general features observed *in vitro:* i.e., lack of expression of classic co-stimulatory molecules and ability to downregulate NK and T-cells proliferation (48, 49), which balances the risk–benefit profile toward being a safe therapy. The generation of DSA are the consequence of indirect allo-recognition of HLAs from allogeneic MSCs by patient APC. As a result, the induction of allo-specific CD4^+^ T cells will activate the HLA-specific-IgG producing B cells (18).

Investigation of DSA induced by ASC was an important focus in the ADMIRE CD study. In ADMIRE CD we devoted efforts to understand the immunogenic impact of ASC in pre-sensitized and naïve patients. The present study sheds light on the persistence and function of DSA, and provides *in vitro* mechanistic insights in HLA Abs and DSA interactions with ASC. Here we confirm that patients can be primed by allo-MHC molecules and mount a DSA response against allogeneic ASC after a single local injection. Pre-sensitization to allo-antigens can result from priming due to the transfusion of blood components (i.e., thrombocytes, leukocytes), organ or cell transplantation, pregnancy, or simply by unspecific cross-reactivity (50). In fact, HLA-specific memory T-cells could be detected in 6–12% of healthy donors, yet >50% of patients on transplant waiting lists (51, 52). Lessons from allogeneic solid-organ and hematopoietic stem cell transplantation indicate that HLA Abs are a major limitation of effective tissue and organ transplantation (34, 53–55). In line with this, we envision that HLA Abs are key contributors to the immediate elimination of allogeneic MSC before they exert modulating effect on inflammation.

The present data demonstrates that a higher proportion of pre-sensitized patients generate and maintain DSA over time, compared with the naïve population. As expected, pre-sensitized patients sharing specificities with the administered product were prone to generate, maintain or increase the DSA titer, indicating a boost effect analogous to a secondary response. However, there is clear evidence showing that pre-existing anti-HLA abs can also lead to a primary allogeneic response when HLA Abs do not share specificities with administered ASC.

Even if it appears not to be a correlation between DSA generation and safety and efficacy of the treatment (24 and Supplementary Figure 1A), the potential impact on a future administration of an allogeneic cell therapy product or transplantation is still unknown. To evaluate the risk-benefit of allogeneic treatment in pre-sensitized patients, the consequences of the presence of HLA Abs *in vivo* should be also evaluated.

Allo-antibodies can bind to HLA and subsequently initiate antibody-dependent cytotoxicity because of their interaction with innate immune cells *via* Fc receptors (56, 57). Complement can also bind Fc region of the HLA Abs antibody resulting in CDC (52, 56, 58). Cancer cells have developed different strategies for CDC evasion, for instance over-expression of mCRPs (29, 59, 60). Our data indicates that to some extent ASC are also well-equipped for complement system evasion. We observed an approximate ~3.49-fold increased expression of CD46 in ASC compared to BM-MSC in pro-inflammatory conditions, suggesting ASC have the ability to induce CD46 expression. The canonical role of CD46 is to bind and inhibit the opsonin factors C3b and C4b and therefore its function has mainly been linked to innate immunity. However, recent investigations have highlighted the role of CD46 in adaptive immunity (61, 62). CD46 has been directly implicated in the modulation of Th1 IFNγ to interleukin-10, switching to and regulating an adaptive T-cell response (61). Such evidence implicates CD46 as playing a pivotal role in immunogenic response, further investigations are required to characterize the extent at which it may be relevant for influencing the immunomodulatory properties of ASC. Finally, we cannot exclude that some other immune-evasive strategies, such as the over-expression of heat-shock proteins or complement inhibitors, contribute to ASC immune-evasive capabilities (29, 63). Several groups are evaluating the modulation of complement evasive molecules to cope with allogeneic MSC cytotoxicity and subsequently prolong MSC persistence *in vivo*. Herein, we propose the examination of CDC/HLA^MFI^ curves and HLA binding kinetics for assessing donors' differential susceptibility to complement dependent toxicity.

The capacity of HLA-I to transduce signals is dependent on the degree of molecular aggregation of the HLA-I molecules which relies on the level of HLA-I expression and HLA antibody titer. In solid organ transplants, high antibody titer induces death of endothelial cells; a low titer promotes their survival and CDC resistance (64, 65). In addition, the migration of monocytes/macrophages in response to Fc-dependent effector factors has also been proposed to have a relevant role (66, 67). In this case, the presence of DSA could potentially also promote monocyte macrophages trafficking toward ASC. We could show though that pre-existing HLA Ab from patients' serum bind and trigger CDC onto ASC *in vitro*; only in the presence of high levels of DSA and when ASC are preactivated could the killing effect of ASC be observed. Although the present data supports darvadstrocel efficacy and long-term DSA clearance in naïve patients, there are outstanding questions that remain unanswered. First, is whether the indirect allorecognition of the foreign HLA molecule could be defined as the consequence of donor-to-patient crosstalk (i.e., NK cells, monocytes, macrophages) and whether the survival and modulatory mechanisms of ASC are causing a delay or reduction of this allo-response. Secondly, it remains to be determined whether MSC elimination is directly associated with a lack of efficacy or, as proposed in other studies, that apoptosis of MSC and the engulfment of phagocytes with apoptotic MSC is in fact essential for MSC modulation (68). These outstanding questions need to be addressed not only in experimental models but also *via* extensive monitoring in clinical studies in which therapeutic efficacy, TEAEs and immunogenicity are interconnected and correlated.

As a summary, we have found that despite allo-ASC generate DSA, those levels are not sufficient to bind ASC and subsequently execute antibody-dependent cytotoxicity *in vitro*. Only particular cases where the number of mismatched *eplets* is extremely high, the DSA titer could potentially be detrimental for ASC survival, in particular if ASC are activated. To reduce the DSA titer, we are inclined to propose donor screening for optimal eligibility rather than donor-to-patient HLA-matching oriented strategies as proposed by others (69). An interesting scheme, similar to what has been proposed for allogeneic induced pluripotent stem cells banks, is the generation of ASC banks from HLA-homozygous donors (70), minimizing the number of allo-antigens exposed to the patient. An interesting approach to minimize immediate rejection in pre-sensitized patients might be the fine-tune modulation of mCRPs such as CD46 in ASC donors. We propose CD46^HIGH^ as a biomarker of CDC resistance that could be used for screening ASC donors suitable for the treatment of pre-sensitized patients and/or facilitating re-treatment options.

## Data Availability

All datasets generated for this study are included in the manuscript and/or the Supplementary Files.

## Ethics Statement

Current study involve use of frozen plasma samples belonging to ADMIRE CD clinical trial. The study was done in accordance with the 2008 Declaration of Helsinki and all relevant international, national, and local rules and regulations (https://www.clinicaltrialsregister.eu/ctr-search/trial/2011-006064-43/results). The protocol was approved by the local ethics committee of participating centers. All patients gave written informed consent before enrolment.

## Author Contributions

OD and AA-V: conceptualization, visualization, and supervision. AA-V, CM-M, CR, BD, RM, PM-C, and MO-V: methodology. AA-V and CM-M: investigation. JC and OD: resources. AA-V, OD, and CM-M: writing. AA-V, OD, WD, EL, JP, CM-M, MO-V, ÁH-M, PM-C, DG-O, and IP: revision. OD: funding resources.

### Conflict of Interest Statement

The authors declare that this study received funding from Tigenix. The funder was involved in the study design, execution, planning, analysis and interpretation. AA-V, CR, BD, RM, PM-C, MO-V, ÁH-M, IP, EL, and OD are employees of and WD is a former employee of Tigenix (Takeda Pharmaceuticals). CM-M and JC were subcontracted by Tigenix for DSA monitoring studies. JP has received consulting fees from TiGenix, Takeda, Abbvie, Arena, Boehringer-Ingelheim, Celgene, Genentech, GSK, Janssen, MSD, Nestlé, Oppilan, Pfizer, Progenity, Roche, and Theravance. DG-O is a member of Advisor Board of TiGenix SAU; is an inventor in a patent “Identification and isolation of multipotent cells from non-osteochondral mesenchymal tissue” (10157355957US), pending to TiGenix; is an inventor in a patent “Use of adipose tissue derived stromal stemcells in treating fistula” (US11/167061), pending to TiGenix; and received consulting fees from Takeda.

## Supplementary Material

Supplementary Figure 1 Effect of DSA generation at W12 and W52 in the proportions of patients with clinical remission (closure of all treated external openings that were draining at baseline despite gentle finger compression). (N) is the number of patients in analysis population and (n) number of patients with observation. There was no clinical remission data for 10 naïve patients which explains why number of clinical remission data-points (43 patients) differs to total number of naïve patients in the study (53 patients) Figure 1A. Percentages were calculated based on HLA Abs status at baseline and patients exhibiting DSA both at W12 and W52. **(B)** DNA from DonA and DonB was purified and tested by LABTypeSSO assay for HLA allele characterization. In bold, we highlight HLA-A allele shared by DonA and DonB.

Supplementary Figure 2 ADMIRE CD plasma samples induce low cytotoxic killing in ASC *in vitro*. Graphs showing normalized percent values of 7-AAD positive ASC in 10 pre-sensitized (upper panels) and 17 *de novo* DSA+ patients (lower panels) before and after INFγ stimulation at the indicated time-points (week 0 and week 12).

Supplementary Figure 3 We correlated MFI values of W6/32 **(A)** and CD46, CD55, and CD59 **(B)** of ASC donors grown in the presence of 3 ng/mL IFNγ for 48h (red dots) or basal conditions (black dots). *P*-values shows slope significance of linear regression. The R-squared value for the significant of the slopes in IFNγ conditions in CD46 and CD55 **(B)** was 0.74 and 0.71, respectively. **(C)** Left image, annotation of human full-length messenger RNA (mRNA) of CD46. Highlighted in blue (crRNA1) and yellow (crRNA2) are the specific guide RNA sequences tested and green are the protospacer adjacent motif (PAM) sites. Lastly, red lines correspond to CAS9 cleavage sites. The right image shows the merged picture of phase contrast and fluorescence PE channel of ASC transfected with ribo-nucleo-protein complex including tracrRNA-ATTO^550^ (red). Scale bar = 20 μm. **(D)** Graph showing CD46 MFI values in parental (black) and CD46^KO^ (dark and light red) ASC. Prior analysis parental and CD46^KO^ ASC were grown in the presence of 3 ng/mL IFNγ for 48 h (IFNγ) or left untreated (basal). *P*-values were determined by the Student's *t*-test.
