# Supplementary figures and images for: Dissecting Allo-Sensitization After Local Administration of Human Allogeneic Adipose Mesenchymal Stem Cells in Perianal Fistulas of Crohn's Disease Patients
Source: Front Immunol. 2019 Jun 14;10:1244. doi: 10.3389/fimmu.2019.01244 (PMC6587893; doi:10.3389/fimmu.2019.01244)

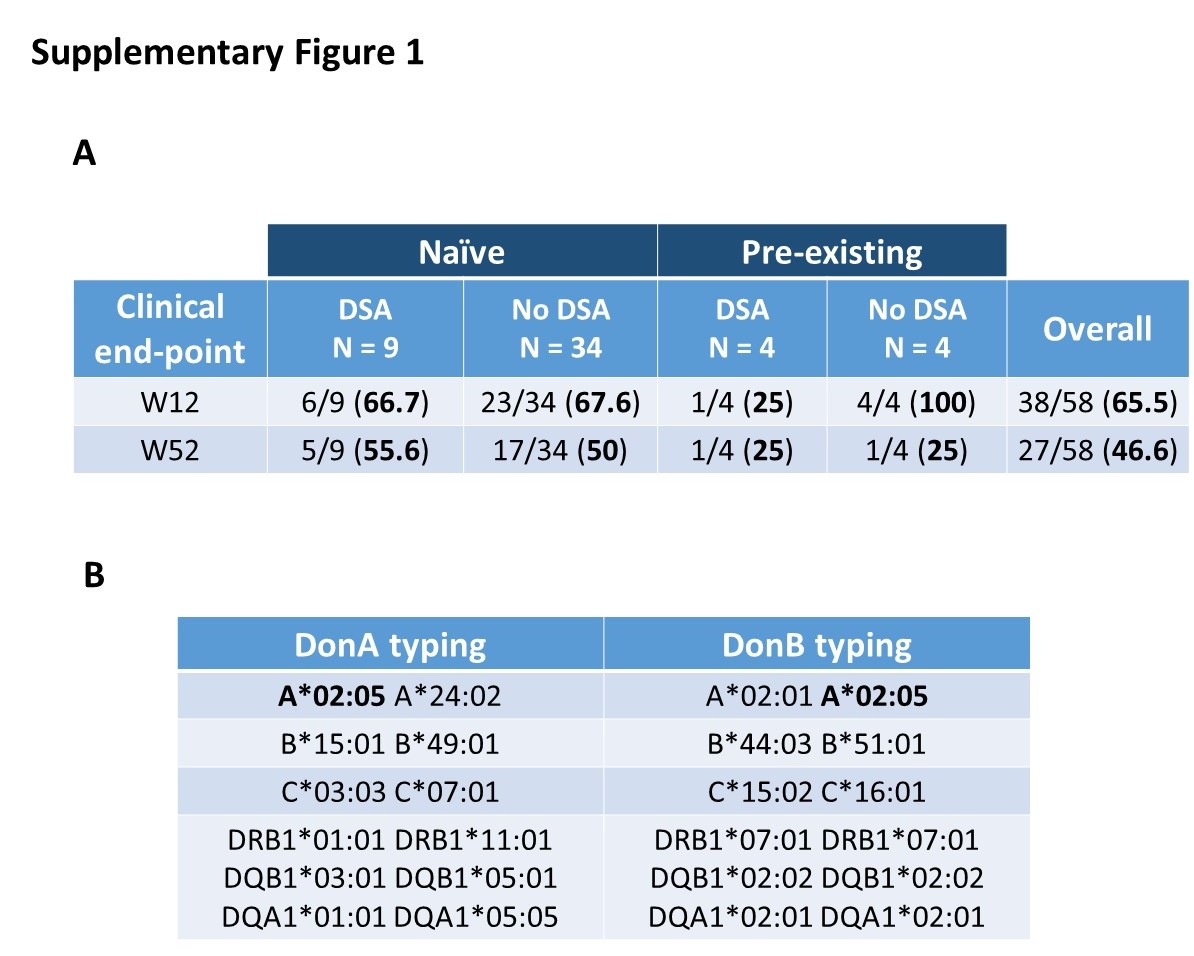

Supplement: Supplementary Figure 1 — Effect of DSA generation at W12 and W52 in the proportions of patients with clinical remission (closure of all treated external openings that were draining at baseline despite gentle finger compression). (N) is the number of patients in analysis population and (n) number of patients with observation. There was no clinical remission data for 10 naïve patients which explains why number of clinical remission data-points (43 patients) differs to total number of naïve patients in the study (53 patients) Figure 1A. Percentages were calculated based on HLA Abs status at baseline and patients exhibiting DSA both at W12 and W52. (B) DNA from DonA and DonB was purified and tested by LABTypeSSO assay for HLA allele characterization. In bold, we highlight HLA-A allele shared by DonA and DonB. [file Image_1.JPEG]

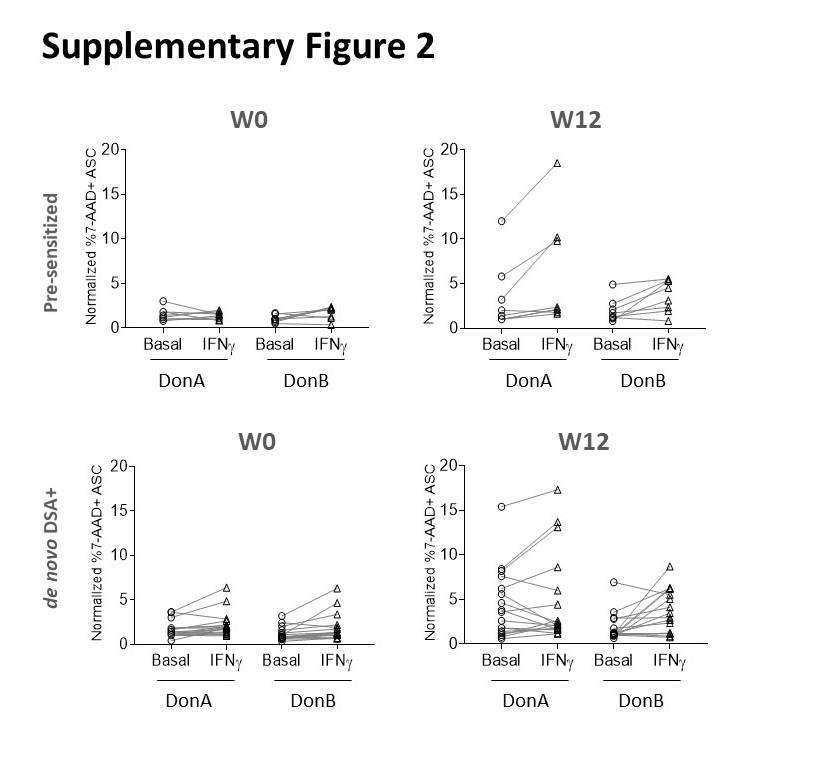

Supplement: Supplementary Figure 2 — ADMIRE CD plasma samples induce low cytotoxic killing in ASC in vitro. Graphs showing normalized percent values of 7-AAD positive ASC in 10 pre-sensitized (upper panels) and 17 de novo DSA+ patients (lower panels) before and after INFγ stimulation at the indicated time-points (week 0 and week 12). [file Image_2.JPEG]

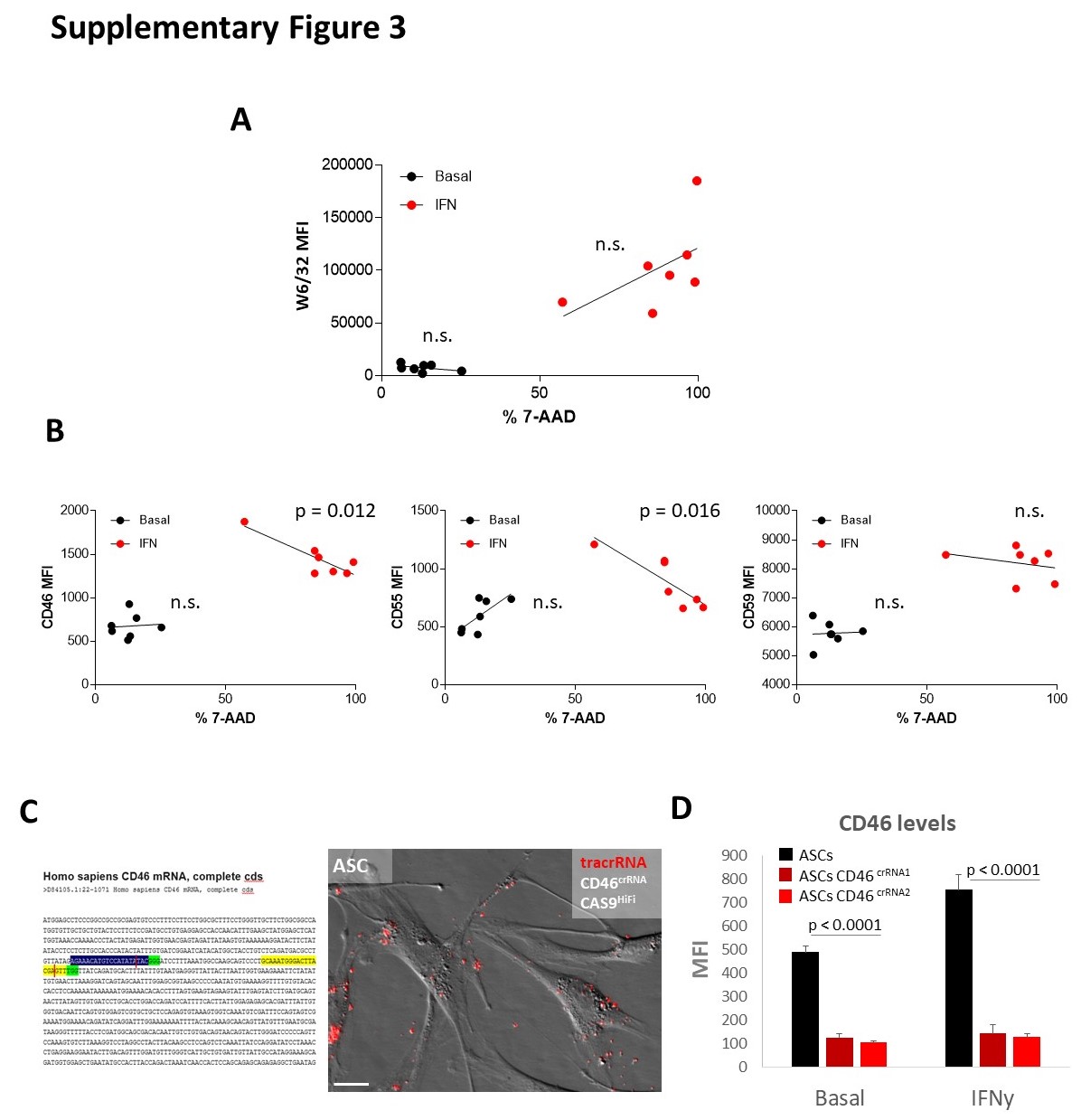

Supplement: Supplementary Figure 3 — We correlated MFI values of W6/32 (A) and CD46, CD55, and CD59 (B) of ASC donors grown in the presence of 3 ng/mL IFNγ for 48h (red dots) or basal conditions (black dots). P-values shows slope significance of linear regression. The R-squared value for the significant of the slopes in IFNγ conditions in CD46 and CD55 (B) was 0.74 and 0.71, respectively. (C) Left image, annotation of human full-length messenger RNA (mRNA) of CD46. Highlighted in blue (crRNA1) and yellow (crRNA2) are the specific guide RNA sequences tested and green are the protospacer adjacent motif (PAM) sites. Lastly, red lines correspond to CAS9 cleavage sites. The right image shows the merged picture of phase contrast and fluorescence PE channel of ASC transfected with ribo-nucleo-protein complex including tracrRNA-ATTO550 (red). Scale bar = 20 μm. (D) Graph showing CD46 MFI values in parental (black) and CD46KO (dark and light red) ASC. Prior analysis parental and CD46KO ASC were grown in the presence of 3 ng/mL IFNγ for 48 h (IFNγ) or left untreated (basal). P-values were determined by the Student's t-test. [file Image_3.JPEG]
